# Exploration of Key Immune-Related Transcriptomes Associated with Doxorubicin-Induced Cardiotoxicity in Patients with Breast Cancer

**DOI:** 10.1007/s12012-023-09806-5

**Published:** 2023-09-08

**Authors:** Daiqin Xiong, Jianhua Yang, Dongfeng Li, Jie Wang

**Affiliations:** Department of Pharmacy, The First Affiliated Hospital of Xinjiang Medical University, Urumqi, 830011 China

**Keywords:** Breast cancer, Doxorubicin, Cardiotoxicity, Immunological genes, Binding affinity

## Abstract

Based on a few studies, heart failure patients with breast cancer were assessed to find potential biomarkers for doxorubicin-induced cardiotoxicity. However, key immune-related transcriptional markers linked to doxorubicin-induced cardiotoxicity in breast cancer patients have not been thoroughly investigated. We used GSE40447, GSE76314, and TCGA BRCA cohorts to perform this study. Then, we performed various bioinformatics approaches to identify the key immune-related transcriptional markers and their association with doxorubicin-induced cardiotoxicity in patients with breast cancer. We found 255 upregulated genes and 286 downregulated genes in patients with doxorubicin-induced heart failure in breast cancer. We discovered that in patients with breast cancer comorbidity doxorubicin-induced cardiotoxicity, the 58 immunological genes are elevated (such as *CPA3, VSIG4, GATA2, RFX2, IL3RA,* and *LRP1*), and the 60 genes are significantly suppressed (such as *MS4A1, FCRL1, CD200, FCRLA, FCRL2,* and *CD79A*). Furthermore, we revealed that the immune-related differentially expressed genes (DEGs) are substantially associated with the enrichment of KEGG pathways, including B-cell receptor signaling pathway, primary immunodeficiency, chemokine signaling pathway, hematopoietic cell lineage, cytokine-cytokine receptor interaction, Toll-like receptor signaling pathway, MAPK signaling pathway, focal adhesion, dilated cardiomyopathy, cell adhesion molecule, etc. Moreover, we discovered that the doxorubicin-induced immune-related genes are crucially involved in the protein–protein interaction and gene clusters. The immune-related genes, including *IFIT5, XCL1, SPIB, BTLA, MS4A1, CD19, TCL1A, CD83, CD200, FCRLA, CD79A, BIRC3,* and *IGF2R* are significantly associated with a poor survival prognosis of breast cancer patients and showed diagnostic efficacy in patients with breast cancer and heart failure. Molecular docking revealed that the survival-associated genes interact with the doxorubicin with appreciable binding affinity. Finally, we validated the expression level of immune-related genes in breast cancer patients-derived cardiomyocytes with doxorubicin-induced cardiotoxicity and found that the level of *RAD9A, HSPA1B, GATA2, IGF2R, CD200, ERCC8,* and *BCL11A* genes are consistently dysregulated. Our findings offered a basis for understanding the mechanism and pathogenesis of the cardiotoxicity caused by doxorubicin in breast cancer patients and predicted the interaction of immune-related potential biomarkers with doxorubicin.

**Supplementary Information:**

The online version contains supplementary material available at 10.1007/s12012-023-09806-5.

## Introduction

Breast cancer is the most frequent cancer in women and is also one of the most common reasons for death from cancer [1]. The highest death rate in women is associated with cardiovascular disease (CVD). Breast cancer treatment has a significant negative impact on cardiovascular health in general. Various risk factors for CVD and breast cancer overlap, such as nutrition, tobacco, and being overweight [2]. Heart failure and breast cancer are both linked to similar risks and morbidities. Receiving multimodal therapy for breast cancer may increase the risk of patients developing heart failure both during and after therapy [3]. Cardiotoxicity is a side effect of cancer treatment that might manifest immediately or take time to manifest [4]. Several cancer treatments, such as anthracyclines, may result in progressive cardiac remodeling as a late outcome of earlier myocyte loss, resulting in heart failure, and even cause momentary cardiac malfunction without lengthy consequences in delayed cardiomyopathy[2, 5]. Cardiotoxicities related to treatment are becoming a growing source of fear for people with breast cancer [6]. A population-based cohort analysis identified the precise frequency of CVD, including heart failure, ischemic heart disease, and arrhythmia in breast cancer patients [6]. Compared to non-cancer individuals, patients who have received extremely cardiotoxic therapies, such as anthracycline medication, are more susceptible to developing CVD [2]. The use of anthracyclines in treating breast cancer emerges from their involvement in enhancing patient survival. Strong evidence confirming the influence of anthracyclines on breast cancer survival has made them a mainstay of breast cancer treatment for decades. Doxorubicin (DOX), an anthracycline, has long been the preferred medication for breast cancer treatment [7]. However, worries about uncommon but severe long-term complications, including cardiotoxicity and cancers, have stoked interest in alternate treatment approaches with suitable toxicity profiles [8]. A prospective cohort study also quantified anthracycline-induced cardiotoxicity in breast cancer patients [9]. Anthracycline therapy has been linked to various forms of cardiotoxicity. After taking a single dose or a course of anthracycline medicine, cardiotoxicity might happen in the first week [10]. Individuals getting a high cumulative dose of doxorubicin, patients at either end of the age spectrum, patients who identify as female, and those with underlying cardiovascular disorders are more likely to develop cardiomyopathy[11, 12]. Doxorubicin induces cardiomyocyte death by regulating the signaling pathways, including autophagy, ferroptosis, necroptosis, pyroptosis, and apoptosis [13]. For example, doxorubicin disrupts the function of transcriptional factors that control lysosomal functioning, causing proteotoxicity, mitochondrial malfunction, and cell death. As a result, the heart is more vulnerable to cardiomyopathic collapse [14].

It is well-known that doxorubicin is a chemotherapeutic agent for various malignancies, but cardiotoxicity is a significant adverse effect. Research disclosed that doxorubicin-based therapy alters the gene expression profile in breast cancer [15]. Deregulating genes involved in autoimmune and inflammatory diseases were linked to increased susceptibility to doxorubicin cardiotoxicity [16]. Cardiotoxicity caused by doxorubicin is linked to altered protein and gene expression, including apoptosis. Additionally, doxorubicin causes the epigenetic markers to be downregulated, which slows down the DNA methylation process [17]. Breast cancer cells treated with doxorubicin exhibit increased nuclear B7-H1 expression and decreased cell surface B7-H1 expression [18]. This group also reported that anthracyclines affect cancer cells and provide a link between immunoresistance and chemoresistance [18]. Doxorubicin generated immunological abnormalities and inflammatory responses via HMGB1, HIF1- and VEGF pathways and was investigated in the progression of cardiovascular disease [19]. Doxorubicin-induced cardiotoxicity is accompanied by the identification of impairment, the stimulation, and the secretion of soluble mediators such cytokines, chemokines, and proteases, as well as immune cells like monocytes, macrophages, and neutrophils [20]. Therefore, several strategies to reduce anthracycline-induced cardiac damage through genetic ways exist. For example, SIRT1 may offer vitally important cardiovascular protection in doxorubicin-induced cardiotoxicity [21]. Through the preservation of mitochondrial homeostasis and reduction of oxidative stress and apoptosis, activation of the AMPK/PGC1 pathway may provide a novel mechanism against acute DOX cardiotoxicity [22]. As mentioned above, the studies suggested that the dysregulated genes and signaling pathways are crucially associated with doxorubicin-induced cardiotoxicity in breast cancer patients. Herein we aimed to identify the key immune-related transcriptional markers linked to doxorubicin-induced cardiotoxicity in patients with breast cancer that have not been thoroughly investigated.

## Materials and Methods

### Datasets

We used GSE40447[23], GSE76314[24], and The Cancer Genome Atlas (TCGA) BRCA cohort in Gene Expression Profiling Interactive Analysis (GEPIA)[25] for this study. The GSE40447 is based on Affymetrix Human Genome U133 Plus 2.0 Array with five breast cancer women with doxorubicin-induced heart failure and 10 breast cancer women without heart failure. Whole blood RNA from women with and without chemotherapy-induced cardiotoxicity was profiled to identify possible biomarkers of sensitivity to heart failure. The GSE76314 is based on the Illumina HiSeq 2000 platform with a gene expression profile of human-induced pluripotent stem cell-derived cardiomyocytes (hiPSC-CMs) from six patients and six controls. We utilized GEPIA, a web-based tool for using the TCGA BRCA cohort. Moreover, we used the TCGA BRCA cohort’s clinical data to identify the association of key genes with survival probability.

### Exploration of Doxorubicin-Induced Differentially Expressed Genes in Patients with Heart Failure and Breast Cancer

We used GSE40447[23] for identifying the differentially expressed genes in women with breast cancer and doxorubicin-induced cardiotoxicity. We identified the differentially expressed genes between the women with doxorubicin-induced heart failure and women without heart failure with breast cancer. We used the R package limma to analyze differential expression in gene expression datasets [26]. The R package limma performs more effectively when analyzing datasets from gene ex-pression profiling. The package includes powerful tools for reading, preprocessing, normalizing, and investigating gene expression data. Proper data preprocessing, nor-malization, and quality control are critical to ensure reliable results. We averaged the multiple values into a single value to compare the groups for several probes of a single-gene. After pre-procession, The chosen datasets underwent base-2 log transformation to achieve normalization. The thresholds of *P*-value 0.05 (Adjusted *P*-value < 0.628) and |Log_2_FC|> 0.585 were selected to determine the significant level. The immune-related genes were retrieved from InnateDB (https://www.innatedb.com/) [27]. Common immune-related encoding genes were found in patients with heart failure brought on by doxorubicin using the online tool “Calculate and design custom Venn diagrams” (http://bioinformatics.psb.ugent.be/webtools/Venn/).

### Investigation of the Gene Ontology (GO) and Pathways

Using the GSEA program, we conducted pathway enrichment analysis of the differentially expressed genes [28]. The GO, Kyoto encyclopedia of genes and genomes (KEGG) pathways, and Reactome (https://reactome.org) pathways are identified as strongly connected with the discovered immune-related genes. Using a cut-off *P*-value of 0.05, we chose the significant GO terms, Reactome pathways, and KEGG pathways. To map the KEGG Pathway, we utilized the Pathview R package [29].

### Construction of Protein–Protein Interaction (PPI) and Identification of the Hub-Immune-Related Genes and Gene Modules

The PPI networks of the essential differentially expressed genes (DEGs) were built using the web program STRING (version v11) [30]. The hub genes were identified using the cytoHubba [31] plugin for Cytoscape. We found genes in the PPI with a mean interaction score of 0.40 and a degree of interaction of 5. Additionally, we used the Cytoscape software’s molecular complex identification (MCODE) plug-in to identify the important gene modules from the initial PPI network [31]. Finally, using the following criteria, we discovered numerous gene modules: N node score Cut-off is 0.2, Haircut is true, K-Core is 2, maximum depth from Seed is 100, and MCODE score is more than 3.0. Besides, we found immune-related hub genes using the “Calculate and build custom Venn diagrams” (http://bioinformatics.psb.ugent.be/webtools/Venn/) tool by comparing the hub nodes with immune-related hub genes and used the Cytoscape (version 3.6.1) [32] program to display the PPI network.

### Survival Analysis of Key DEGs by Using the GEPIA

Patients with breast cancer underwent a comparison of their overall survival (OS) and disease-free survival (DFS). Kaplan–Meier survival curves were employed to display the differences in survival rates between the high- and low-expression groups. Based on the median expression value of each gene (high-expression group > median > low-expression group), we divided the breast cancer patients into two groups. Using the GEPIA [25] databases, all DEGs in the TCGA BRCA cohort were examined for their survival relevance. When comparing the survival between the two groups, a *P*-value for Cox regression of 0.05 or lower was regarded as significant.

### Evaluation of Diagnostic Efficacy of Immune-Related Hub Genes

The area under the ROC curve (AUC) was utilized to analyze the effectiveness of important genes for discriminating heart failure patients from control samples, and receiver operating characteristic (ROC) curves were computed and visualized to assess the diagnostic value of the immune-related hub genes [33]. An individual gene’s ability to distinguish between heart failure patients and control samples increases with its AUC value. The study defined a significant diagnostic factor as an AUC > 0.5 for the selected hub gene [34].

### Molecular Docking Of Doxorubicin with Key Genes

The drug was submitted to a molecular docking study after being downloaded as the 3-dimensional conformer of doxorubicin (PubChem CID: 31,703). The RCSB Protein Data Bank (RCSB PDB) (https://www.rcsb.org/) was used to download hub proteins that are related to the survival prognosis of breast cancer patients (Table 1). Proteins were created with the aid of Discovery Studio (https://3ds.com/products-services/biovia/products). The complex proteins initially lost all of their ligands and water molecules. Molecular docking research was carried out using the virtual screening program PyRx (https://pyrx.sourceforge.io/).

**Table 1:** List of proteins for molecular docking with doxorubicin

SL	Gene	Protein ID	Link	References
1.	*XCL1*	2HDM	https://www.rcsb.org/structure/2hdm	[57]
2.	*CD19*	6AL5	https://www.rcsb.org/structure/6al5	[58]
3.	*CD83*	5MJ0	https://www.rcsb.org/structure/5mj0	[59]
4.	*BIRC3*	2UVL	https://www.rcsb.org/structure/2uvl	[60]
5.	*TCL1A*	1JSG	https://www.rcsb.org/structure/1JSG	[61]
6.	*FCRLA*	4HWN	https://www.rcsb.org/structure/4HWN	NA
7.	*IFIT5*	3ZGQ	https://www.rcsb.org/structure/3ZGQ	[62]
8.	*BTLA*	2AW2	https://www.rcsb.org/structure/2AW2	[63]
9.	*MS4A1*	6Y9A	https://www.rcsb.org/structure/6Y9A	[64]
10.	*CD79A*	7XT6	https://www.rcsb.org/structure/7XT6	[65]
11.	*IGF2R*	2V5N	https://www.rcsb.org/structure/2v5n	[66]

### Expression Validation of Key Hub Genes in an Independent Cohort Dataset

We used a separate cohort, GSE76314 (*n* = 12), to verify the expression levels of the top 10 hub genes [24]. In the GSE76314, gene expression was evaluated between the control and hiPSC-CM generated from patients following exposure to 1uM doxorubicin for 24 h. We utilized the R program limma to examine differential expression in gene expression datasets [26]. We averaged the multiple values into single values to compare the groups when using numerous probes for the same gene. The thresholds of *P*-value < 0.05 and |LogFC| (fold change) > 0.45 were selected to determine the significant level of DEGs.

## Results

### Exploration of Key Transcriptional Signatures in Patients with Doxorubicin-Induced Heart Failure and Breast Cancer

**Fig. 1:**
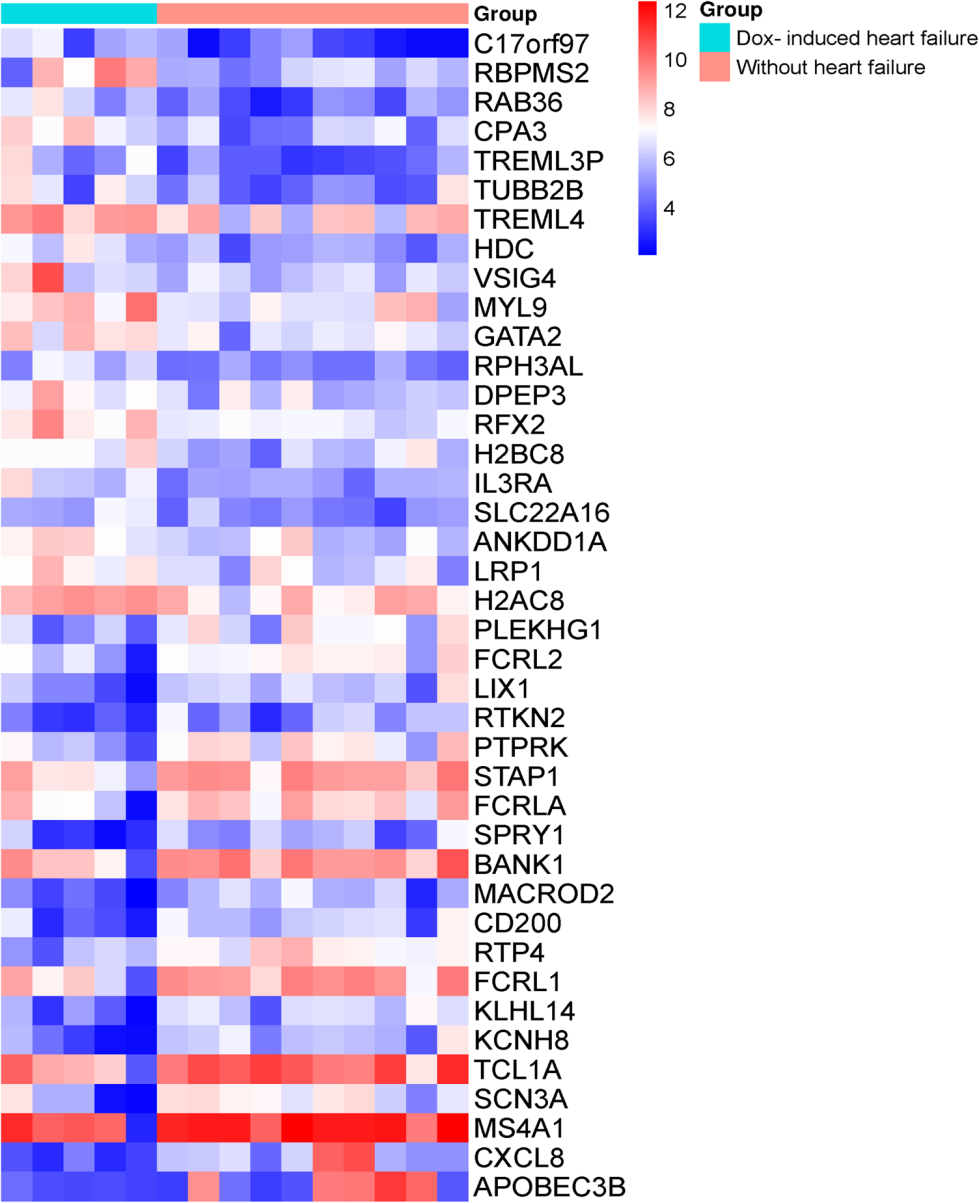
A heat map for each patient showed the top 20 upregulated and top 20 downregulated differentially expressed genes

We compared the transcriptomic signatures between patients with breast cancer and whether treated with doxorubicin. We found that the transcriptomes are differentially expressed between these two groups. We identified the 514 differentially expressed genes in patients with doxorubicin-induced heart failure and breast cancer, including 255 upregulated genes (Supplementary Table S1) and 286 downregulated genes (Supplementary Table S2). Based on the highest Log2FC with *P*-value < 0.05, the top 20 gene are *C17orf97, RBPMS2, RAB36, CPA3, TREML3P, TUBB2B, TREML4, HDC, VSIG4, MYL9, GATA2, RPH3AL, DPEP3, RFX2, H2BC8, IL3RA, SLC22A16, ANKDD1A, LRP1,* and *H2AC8* (Table 2). Similarly, based on the lowest Log2FC with *P*-value < 0.05, the top 20 downregulated genes are *APOBEC3B, CXCL8, MS4A1, SCN3A, TCL1A, KCNH8, KLHL14, FCRL1, RTP4, CD200, MACROD2, BANK1, SPRY1, FCRLA, STAP1, PTPRK, RTKN2, LIX1, FCRL2,* and *PLEKHG1* (Table 3)*.* A heat map for each patient showed the top 20 upregulated and top 20 downregulated differentially expressed genes individually (Fig. 1).

**Table 2:** The top 20 upregulated genes in patients with doxorubicin-induced heart failure and breast cancer

Entrez ID	Log2FC	*P*-value	Symbols	Name
400566	2.07	7.35E-03	*C17orf97*	Chromosome 17 open reading frame 97
348093	2.00	1.61E-02	*RBPMS2*	“RNA binding protein, mRNA processing factor 2”
9609	1.96	3.17E-03	*RAB36*	“RAB36, member RAS oncogene family”
1359	1.90	9.25E-03	*CPA3*	Carboxypeptidase A3
340206	1.86	7.37E-03	*TREML3P*	“Triggering receptor expressed on myeloid cells like 3, pseudogene”
347733	1.64	4.96E-02	*TUBB2B*	Tubulin beta 2B class IIb
285852	1.49	3.62E-02	*TREML4*	Triggering receptor expressed on myeloid cells like 4
3067	1.45	5.18E-03	*HDC*	Histidine decarboxylase
11326	1.44	3.92E-02	*VSIG4*	V-set and immunoglobulin domain containing 4
10398	1.40	2.69E-02	*MYL9*	Myosin light chain 9
2624	1.37	1.25E-02	*GATA2*	GATA binding protein 2
9501	1.36	2.53E-03	*RPH3AL*	Rabphilin 3A like (without C2 domains)
64180	1.36	1.81E-02	*DPEP3*	Dipeptidase 3
5990	1.33	1.42E-03	*RFX2*	Regulatory factor X2
8339	1.30	1.71E-02	*H2BC8*	H2B clustered histone 8
3563	1.29	3.31E-03	*IL3RA*	Interleukin 3 receptor subunit alpha
85413	1.24	1.18E-02	*SLC22A16*	Solute carrier family 22 member 16
348094	1.23	1.64E-02	*ANKDD1A*	Ankyrin repeat and death domain containing 1A
4035	1.23	3.58E-02	*LRP1*	LDL receptor-related protein 1
3012	1.21	1.98E-02	*H2AC8*	H2A clustered histone 8

**Table 3:** The top 20 downregulated genes in patients with doxorubicin-induced heart failure and breast cancer

Entrez ID	Log2FC	*P*-value	Symbols	Name
9582	− 3.18	4.83E-02	*APOBEC3B*	Apolipoprotein B mRNA editing enzyme catalytic subunit 3B
3576	− 3.01	8.18E-03	*CXCL8*	C-X-C motif chemokine ligand 8
931	− 2.43	4.13E-02	*MS4A1*	Membrane spanning 4-domains A1
6328	− 2.32	1.28E-02	*SCN3A*	Sodium voltage-gated channel alpha subunit 3
8115	− 2.27	1.81E-02	*TCL1A*	T-cell leukemia/lymphoma 1A
131096	− 2.24	2.92E-03	*KCNH8*	Potassium voltage-gated channel subfamily H member 8
57565	− 2.17	2.98E-03	*KLHL14*	Kelch-like family member 14
115350	− 2.09	1.06E-02	*FCRL1*	Fc receptor like 1
64108	− 2.03	2.96E-04	*RTP4*	Receptor transporter protein 4
4345	− 1.98	1.40E-02	*CD200*	CD200 molecule
140733	− 1.92	6.00E-03	*MACROD2*	Mono-ADP ribosylhydrolase 2
55024	− 1.87	2.36E-02	*BANK1*	B-cell scaffold protein with ankyrin repeats 1
10252	− 1.86	1.54E-02	*SPRY1*	Sprouty RTK signaling antagonist 1
84824	− 1.83	3.52E-02	*FCRLA*	Fc receptor like A
26228	− 1.76	4.78E-03	*STAP1*	Signal transducing adaptor family member 1
5796	− 1.75	1.47E-02	*PTPRK*	Protein tyrosine phosphatase receptor type K
219790	− 1.75	1.05E-02	*RTKN2*	Rhotekin 2
167410	− 1.73	1.44E-02	*LIX1*	Limb and CNS expressed 1
79368	− 1.70	1.69E-02	*FCRL2*	Fc receptor like 2
57480	− 1.70	2.22E-02	*PLEKHG1*	Pleckstrin homology and RhoGEF domain containing G1

**Fig. 2:**
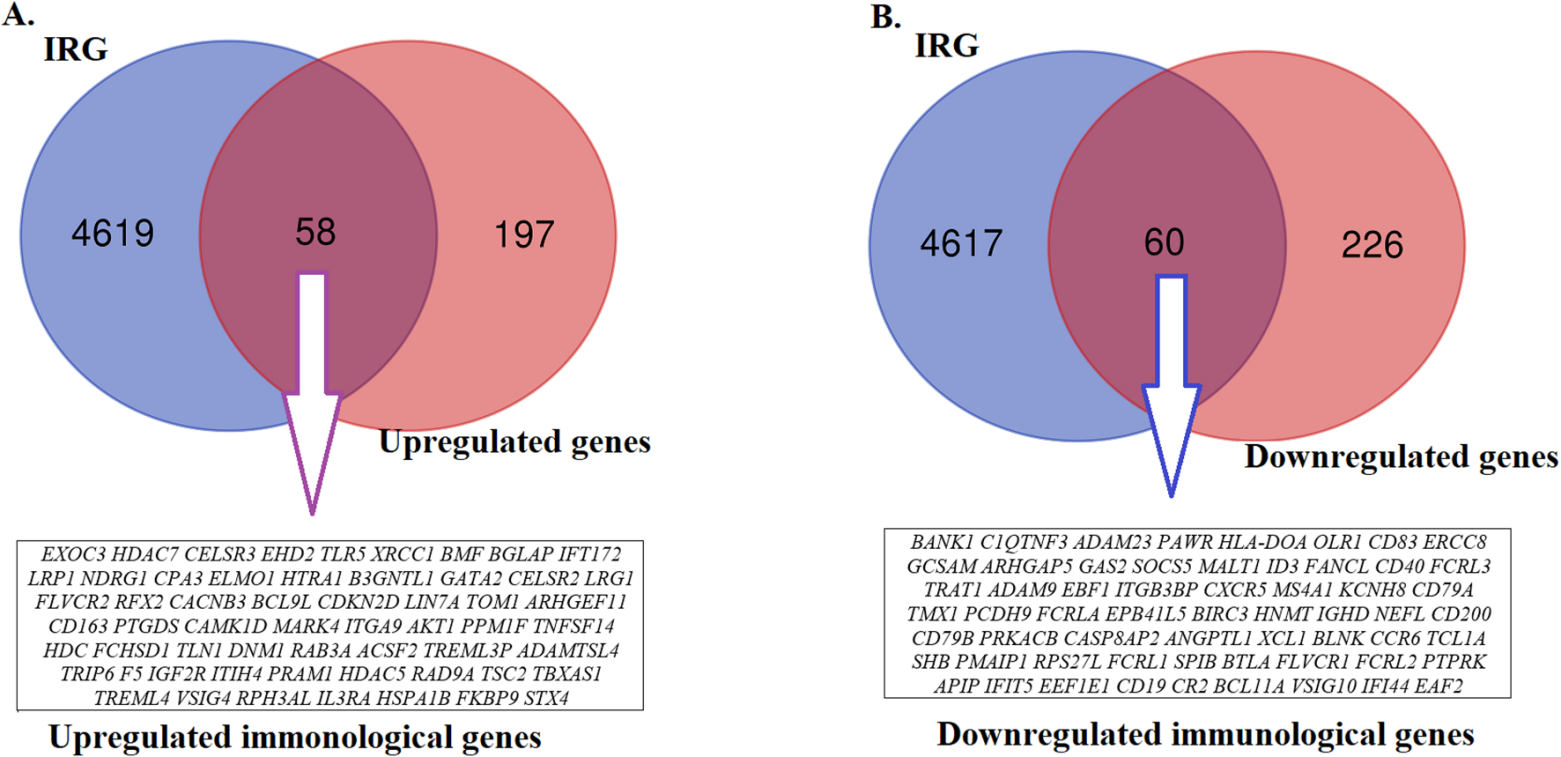
The 58 immunological genes are upregulated (**A**), and the 60 genes are downregulated (**B**) in patients with doxorubicin-induced heart failure and breast cancer. IRG: immunologically related genes

Doxorubicin-induced immunological abnormalities and predicted cardiotoxicity in breast cancer patients are connected with immune-related genes and biomarkers [19, 35]. When DOX causes cardiomyocyte damage, *HMGB1, HIF-1,* and *VEGF* are significant regulators that work through several mechanisms [19]. Moreover, Li-Rong Yu used multiple immunoassays to find proteins that had dramatically altered plasma levels in breast cancer patients receiving cardiotoxic DOX-based chemotherapy, and the immune-related proteins were discovered before DOX administration or at low dosages. Hence, they may be useful as indicators of cardiotoxicity [35]. Therefore, we identified differentially expressed immune-related genes in patients with doxorubicin-induced heart failure and breast cancer. First, we downloaded the immunological-related genes from the InnateDB database (https://www.innatedb.com/) (Supplementary Table S3) and compared these immune-related genes with DEGs significantly differentiated in patients with doxorubicin-induced heart failure and breast cancer. Interestingly, we found that 58 immunological genes were upregulated, and 60 genes were downregulated in breast cancer patients with doxorubicin-induced heart failure (Fig. 2). Based on the highest Log2FC, the top 10 upregulated immunological-related genes are *CPA3, TREML3P, TREML4, HDC, VSIG4, GATA2, RPH3AL, RFX2, IL3RA,* and *LRP1.* Similarly, based on the lowest Log2FC, the top 10 immunological-related genes are *MS4A1, TCL1A, KCNH8, FCRL1, CD200, BANK1, FCRLA, PTPRK, FCRL2,* and *CD79A*.

### Functional Enrichment Analysis of Immunological-Related Genes in Patients with Doxorubicin-Induced Heart Failure and Breast Cancer

**Fig. 3:**
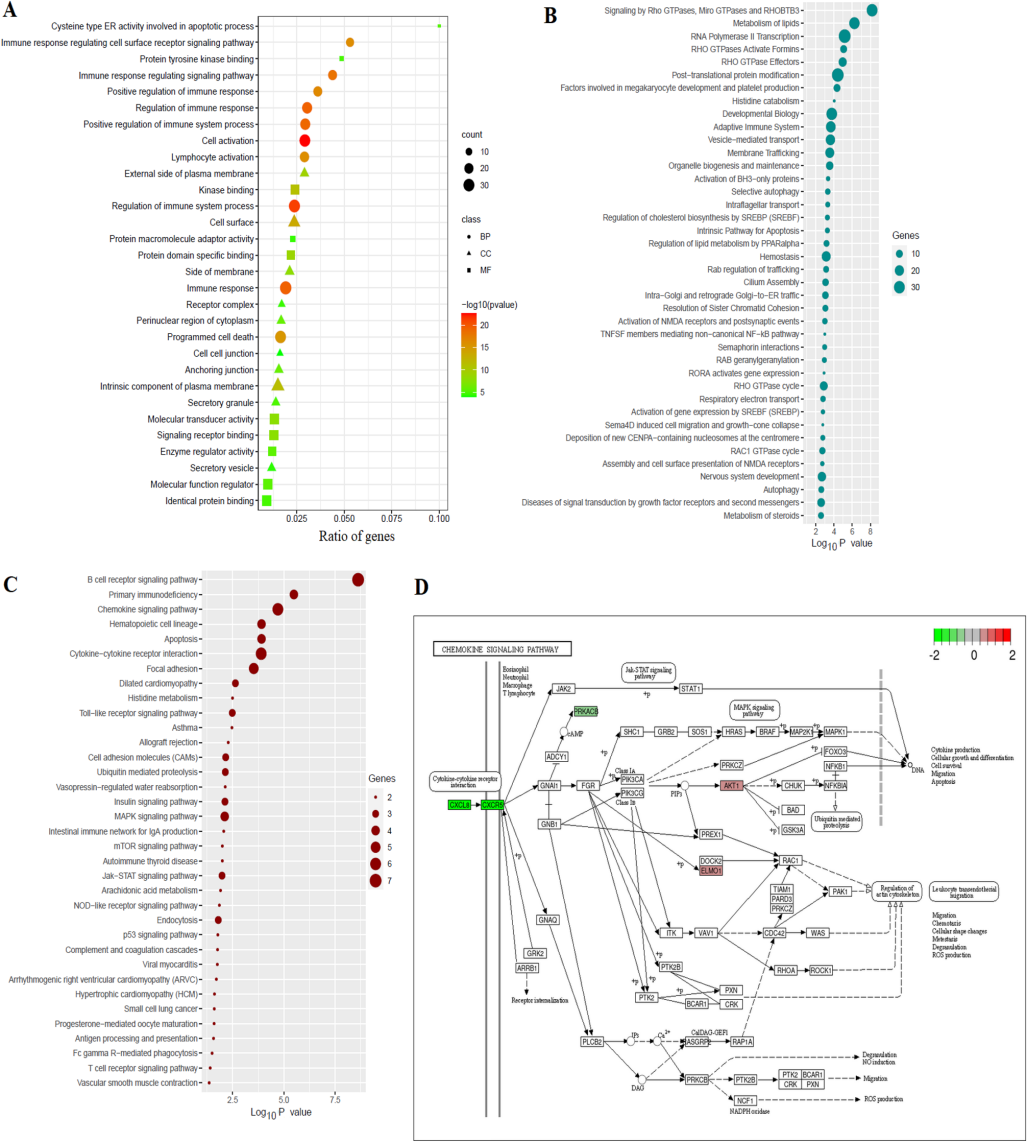
Functional enrichment analysis of immunological-related genes in patients with doxorubicin-induced heart failure and breast cancer. **A** The top enriched biological processes, cellular components, and molecular functions** B** The significantly enriched top Reactome pathways** C** The significantly enriched KEGG pathways **D** The involvement of genes in the chemokine signaling pathway

We found that the immunological-related deregulated genes are significantly associated with the enrichment of GOs. The top enriched biological processes, cellular components, and molecular functions are illustrated in Fig. 3A. Some of the top biological processes are cell activation, regulation of immune system process, immune response, regulation of immune response, positive regulation of immune system process, immune response regulating signaling pathway, and positive regulation of immune response (Fig. 3A and Supplementary Table S4). Also, the top enriched cellular components are the cell surface, an intrinsic component of the plasma membrane, the external side of the plasma membrane, the side of the membrane, anchoring junction, perinuclear region of cytoplasm, secretory granule, receptor complex, secretory vesicle, and cell–cell junction (Fig. 3A and Supplementary Table S5). Moreover, we revealed the enriched molecular functions, including kinase binding, protein domain-specific binding, molecular transducer activity, signaling receptor binding, enzyme regulator activity, and molecular function regulator (Fig. 3A and Supplementary Table S6). The GOs analysis indicated that the deregulated genes are associated with immunological activities via binding with biological membranes to convey the signaling cascades. Then, we identified the enriched Reactome pathways associated with immunological-related dysregulated genes (Fig. 3B and Supplementary Table S7). The enriched Reactome pathways are mainly involved with immunity (such as adaptive immune system, TNF receptor superfamily (TNFSF) members mediating non-canonical NF-kB pathway, chemokine receptors bind chemokines, innate immune system, cytokine signaling in the immune system), metabolism (such as metabolism of lipids, histidine catabolism, and metabolism of steroids, arachidonic acid metabolism, Fatty acid metabolism, and Triglyceride metabolism), and cellular development and signaling cascades (such as signaling by Rho GTPases, Miro GTPases, and RHOBTB3, developmental biology, vesicle-mediated transport, membrane Trafficking, organelle biogenesis and maintenance, activation of BH3-only proteins, selective autophagy, intraflagellar transport, the intrinsic pathway for apoptosis, and cell Cycle) (Fig. 3B and Supplementary Table S7). Furthermore, we revealed that the KEGG pathways are significantly enriched and associated with immunological-related deregulated genes (Fig. 3C). These KEGG pathways are mainly involved with immunological responses (such as B-cell receptor signaling pathway, primary immunodeficiency, chemokine signaling pathway, hematopoietic cell lineage, cytokine-cytokine receptor interaction, Toll-like receptor signaling pathway, allograft rejection, the intestinal immune network for IgA production) and cellular signaling (such as apoptosis, insulin signaling pathway, MAPK signaling pathway, focal adhesion, dilated cardiomyopathy, cell adhesion molecules (CAMs), ubiquitin-mediated proteolysis, and vasopressin-regulated water reabsorption) (Fig. 3C). Various genes are associated with the enrichment of these pathways. For example, *AKT1, PRKACB, CXCL8, CCR6, XCL1, CXCR5,* and *ELMO1* genes regulate the chemokine signaling pathway (Fig. 3D). The doxorubicin-induced deregulated genes are crucially associated with regulating the immune system and cellular signaling in heart failure patients with breast cancer.

### Immunological-Related Genes Are Involved in PPI Networks and Gene Clusters

**Fig. 4:**
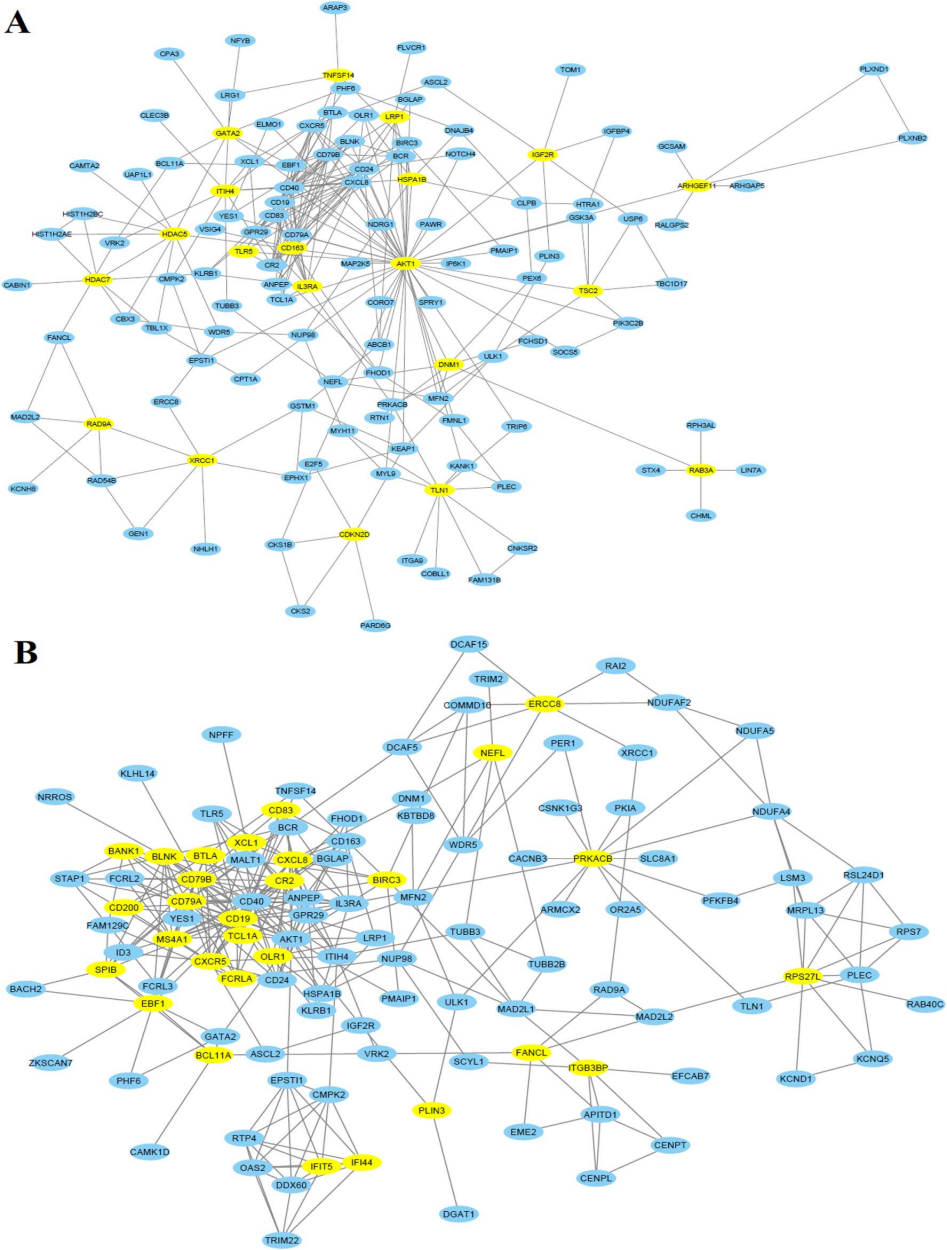
Immunological-related genes are involved in PPI networks. **A** The twenty upregulated immune-related hub genes are involved in the interaction with other DEGs.** B** The twenty-nine downregulated immune-related hub genes are involved in the interaction with other DEGs. Yellow nodes are immune-related hub genes; other nodes are their interacted nodes extracted from the original PPI networks

We inputted all significantly deregulated genes into the STRING tool for identifying the protein–protein interactions (PPI) of these genes. The PPI network information was downloaded and imported to the Cytoscape software for visualizing and identifying the hub genes. We identified hub genes from the PPI network using the Cytoscape plug-in cytoHubba. We applied the medium confidence score (confidence score > 0.40) and degree of interaction equal greater than 5 for identifying the hub genes. Interestingly, 406 genes are involved in the PPI network (Supplementary Table S8). Based on the degree of interaction, we revealed the 114 hub genes (degree ≥ 5) in the PPI network (Supplementary Table S8). Using the Venn diagram, we identified the twenty upregulated and twenty-nine downregulated immune-related hub genes. The twenty upregulated immune-related hub genes are *DNM1, CDKN2D, ARHGEF11, CD163, TLN1, RAB3A, XRCC1, RAD9A, TNFSF14, HSPA1B, TSC2, GATA2, AKT1, ITIH4, LRP1, TLR5, IL3RA, IGF2R, HDAC5,* and *HDAC7*. In addition, the 29 downregulated immune-related hub genes are *ERCC8, CXCL8, IFIT5, XCL1, CD79B, SPIB, BLNK, PRKACB, CR2, FANCL, BTLA, MS4A1, RPS27L, BANK1, CD40, CD19, OLR1, NEFL, IFI44, TCL1A, CD83, CD200, FCRLA, CD79A, EBF1, ITGB3BP, BIRC3, CXCR5,* and *BCL11A.* Then, we extracted the PPI network based on twenty upregulated immune-related hub genes (Fig. 4A). Also, we constructed the PPI network based on twenty-nine downregulated immune-related hub genes (Fig. 4B). It indicates that immune-related deregulated hub genes critically regulate cellular signaling via protein–protein interaction.

**Fig. 5:**
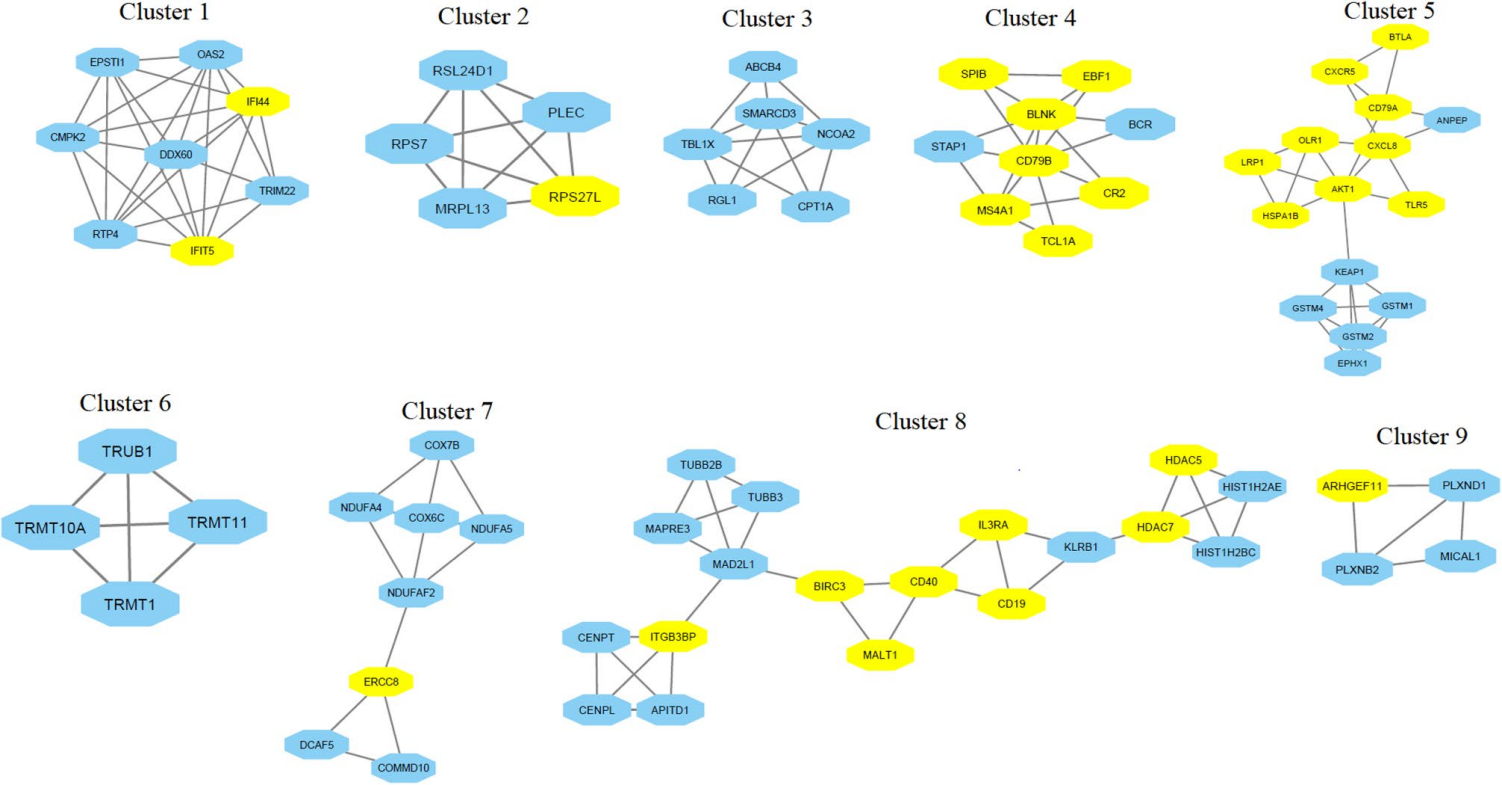
Immunological-related genes are involved in the gene clusters identified using the MCODE algorithm. Yellow nodes are immune-related genes, and other nodes are their interacted nodes in the gene clusters

Furthermore, we identified the key gene clusters from the original PPI network using the Cytoscape plug-in MCODE algorithm. Based on the MCODE score, we identified the nine gene clusters (Fig. 5). Interestingly, we found that the doxorubicin-induced deregulated immunological-related genes are involved in the seven gene clusters (Fig. 5). Cluster 1 involves *IFIT5* and *IFI44* immunological-related genes (Fig. 5). Similarly, *TCL1A, CD79B, MS4A1, EBF1, BLNK, CR2*, and *SPIB* immunological-related genes are involved in cluster 4 (Fig. 5). It indicates that the doxorubicin-induced deregulated immunological-related genes are associated with the signaling cascades through involvement in the gene clusters. Altogether, we identified the nine gene clusters from the original PPI network and we revealed that immune-related genes are involved in the PPI network of these gene clusters. It suggests that the PPI network and gene clusters may be associated with the pathogenesis of doxorubicin-induced cardiotoxicity in breast cancer patients.

### Doxorubicin-Induced Immunological-Related Hub Genes Are Associated with Poor Clinical Outcomes in Breast Cancer Patients

**Fig. 6:**
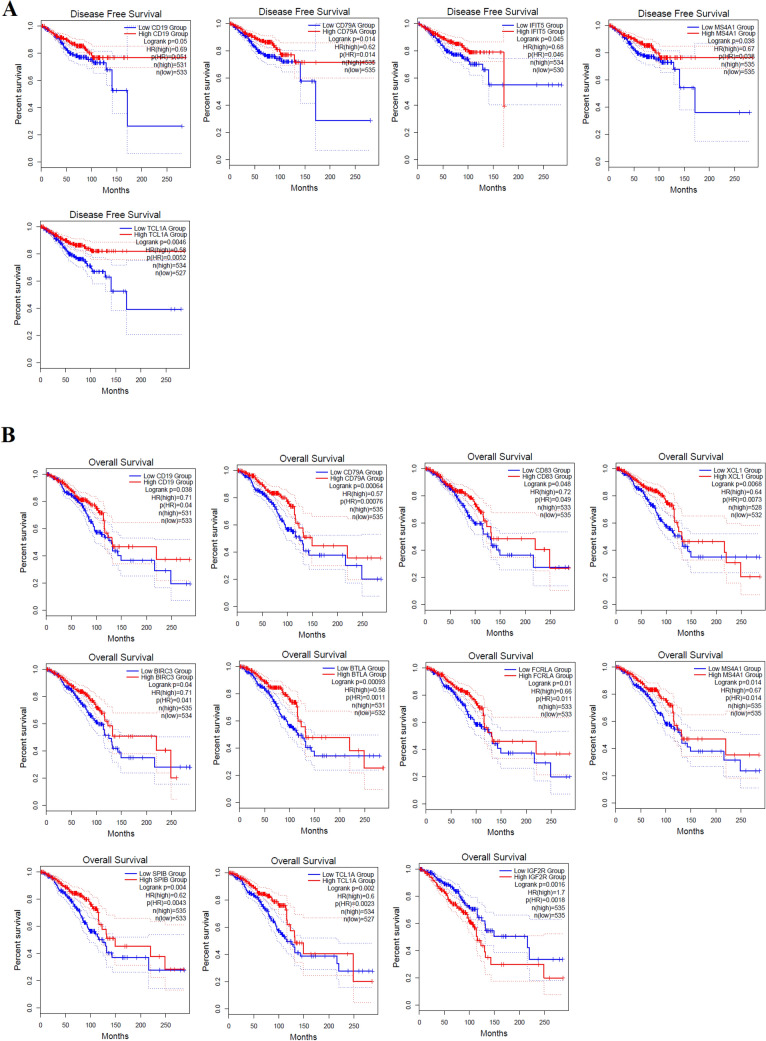
Doxorubicin-induced deregulated immunological-related hub genes are associated with poor clinical outcomes in breast cancer patients. We divided the breast cancer patients into two groups by utilizing the median expression value of individual genes (high: high-expression grou*p* > median > low: low-expression group). **A** The disease-free survival time is shorter in the low-expression group of *CD19, CD79A, IFIT5, MS4A1,* and *TCL1A* genes. **B** Breast cancer patients with the two expression groups of the genes *CD19, CD79A, CD83, XCL1, BIRC3, BTLA, FCRLA, MS4A1, SPIB, TCL1A,* and *IGF2R* had a low overall survival rate

Since doxorubicin‐induced heart failure might be critical for patient survival in breast cancer [36], we identified the survival-associated genes deregulated in doxorubicin-induced heart failure patients with breast cancer. We used GEPEA to investigate the survival prognosis of identified hub genes. Patients with breast cancer who had low levels of expression of the genes *CD19, CD79A, IFIT5, MS4A1,* and *TCL1A* had shorter disease-free survival times (Fig. 6A). Patients with breast cancer had a lower overall survival rate when certain genes, including *CD19, CD79A, CD83, XCL1, BIRC3, BTLA, FCRLA, MS4A1, SPIB, TCL1A,* and *IGF2R,* are expressed at different levels (Fig. 6B). In addition, we revealed that the higher expression group of the *IGF2R* gene is significantly associated with the poor survival of breast cancer patients (Fig. 6). It demonstrated that the upward or downward trend of the expression of these immunological genes is associated with poor clinical outcomes in breast cancer patients, suggesting that the expression of specific immune genes is not only related to doxorubicin-induced cardiotoxicity but may also be an essential risk factor affecting the clinical prognosis of breast cancer patients.

### Survival-Associated Hub Genes Are Associated with Diagnostic Efficacy

**Fig. 7:**
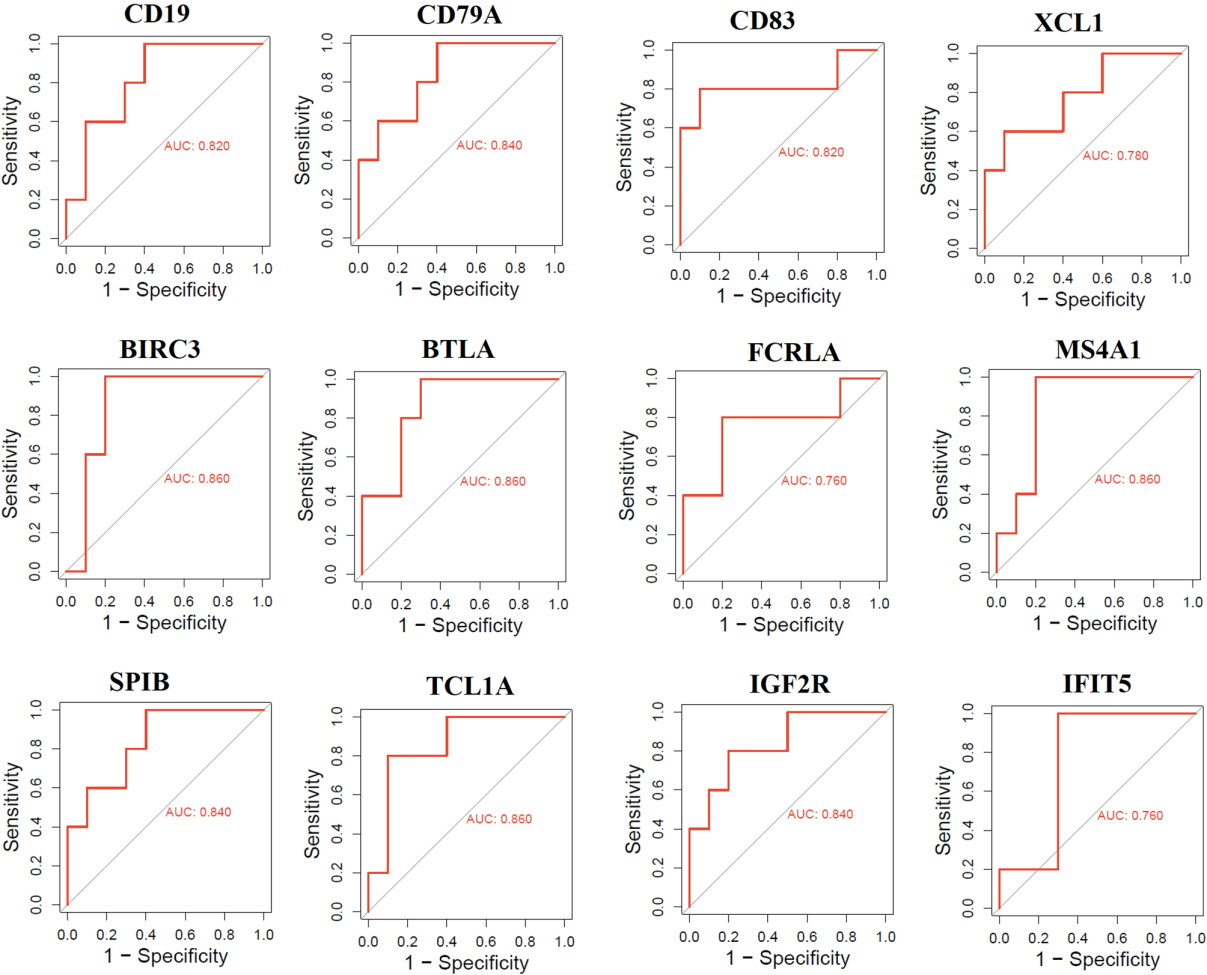
Survival-associated hub genes, including *IFIT5, XCL1, SPIB, BTLA, MS4A1, CD19, TCL1A, CD83, CD200, FCRLA, CD79A,* and *BIRC3* showed diagnostic efficacy between the breast cancer woman with heart failure and without heart failure. The area under the ROC curve (AUC) was utilized to analyze the effectiveness of important genes and receiver operating characteristic (ROC) curves were computed and visualized by utilizing the pROC R package

These immune-related prognostic hub genes (*IFIT5, XCL1, SPIB, BTLA, MS4A1, CD19, TCL1A, CD83, CD200, FCRLA, CD79A,* and *BIRC3*) may be useful for diagnosis. We used GSE40447 to test our hypothesis, and the findings showed that *IFIT5, XCL1, SPIB, BTLA, MS4A1, CD19, TCL1A, CD83, CD200, FCRLA, CD79A,* and *BIRC3* had great diagnostic value for whole blood of heart failure-related breast cancer patients and control sample (Fig. 7).

### Molecular Docking of Prognostic Genes

**Fig. 8:**
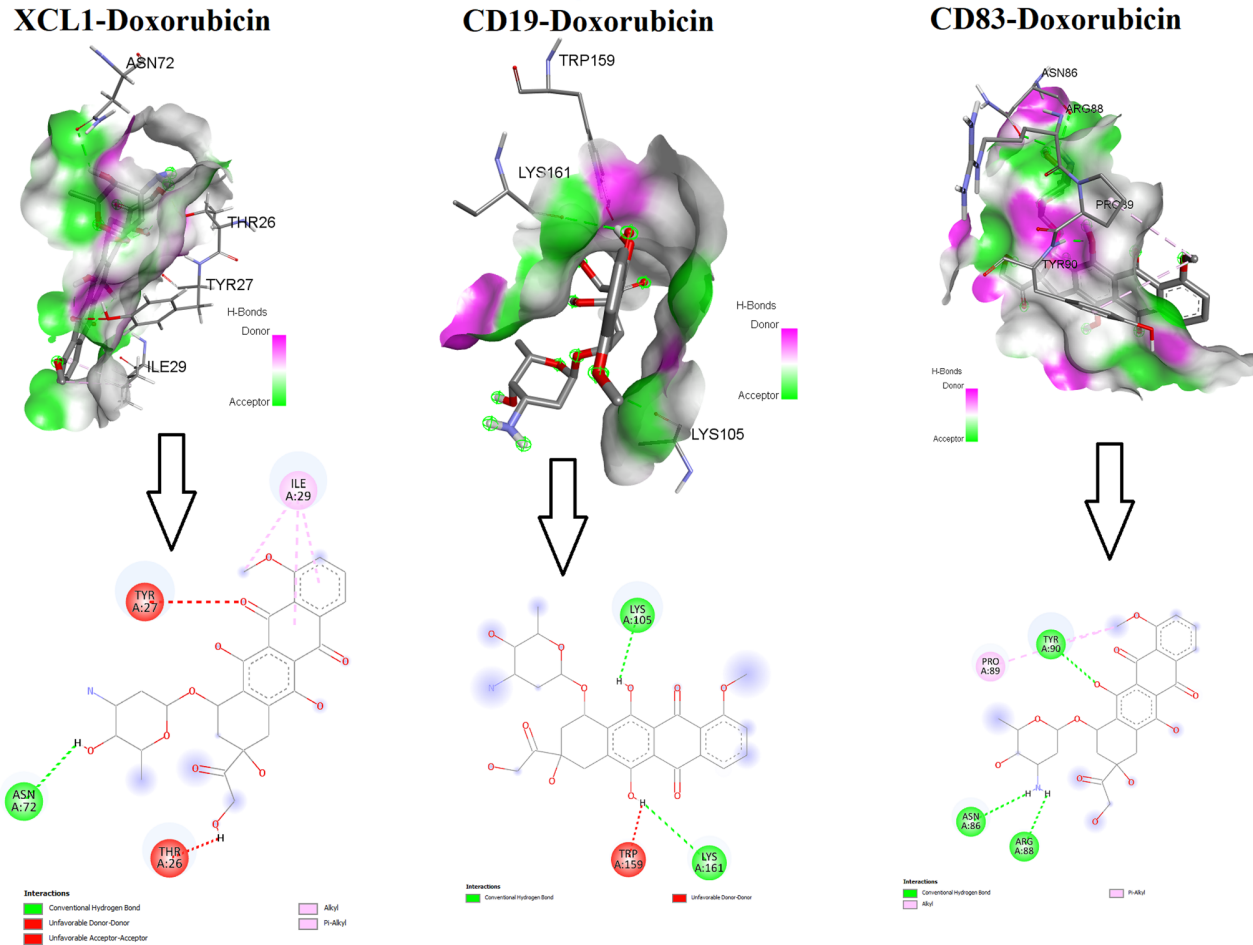
The 3-dimensional and 2-dimensional interactions of the CD19, XCL1, and CD83 protein products with doxorubicin. The RCSB Protein Data Bank (https://www.rcsb.org/) was used to download the protein structures. Protein preparation was carried out with the aid of Discovery Studio (https://3ds.com/products-services/biovia/products). The PyRx tool (https://pyrx.sourceforge.io/) was employed for the molecular docking study

We used molecular docking to identify doxorubicin’s interaction with key survival-associated genes. We found that doxorubicin interacts with the protein product of *IFIT5, XCL1, BTLA, MS4A1, CD19, TCL1A, CD83, CD200, FCRLA, CD79A, IGF2R,* and *BIRC3* genes with an appreciable binding affinity (binding affinity <  − 0.6). We identified that the amino acid residues protein product of CD19 (6AL5), including LYS A: 105, LYS A: 161, and TRP A: 159 interacted with doxorubicin (Fig. 8). Also, the ILE A: 29, TRP A: 27, ASN A: 72, and THR A: 26 amino acid residues of the protein product of XCL1 (2HDM) interacted with doxorubicin (Fig. 8). Moreover, the amino acid residues TYR A: 90, PRO A: 89, ASN A: 86, and ARG A: 88 of the protein product of CD83 (5MJ0) interacted with doxorubicin (Fig. 8). The gene name, protein data bank ID, binding affinity, and interacted amino acid residues are tabulated in Table 4.

**Table 4:** The survival-associated genes, protein data bank ID, binding affinity with doxorubicin, and interacted amino acid residues with doxorubicin

SL	Gene	Protein ID	Binding affinity	Interacted amino acid residues
1	CD19	6AL5	− 6.5	LYS A: 105, LYS A: 161, TRP A: 159
2	XCL1	2HDM	− 7.7	ILE A: 29, TRP A: 27, ASN A: 72, THR A: 26
3	CD83	5MJ0	− 6.5	TYR A: 90, PRO A: 89, ASN A: 86, ARG A: 88
4	BIRC3	2UVL	− 6.5	ARG A: 318,ASP A: 296, CYS A: 294, THR A: 246
5	TCL1A	1JSG	− 6.3	TRP A: 19, LEU A: 27, MET A: 62, ILE A: 74
6	FCRLA	4HWN	− 6.0	GLN A: 272, ALA A: 188, LYS A: 268, GLN A: 269, TRP A: 267
7	IFIT5	3ZGQ	− 8.3	ASP A:189, GLY A:153, ARG A: 186, TYR A:156, ASP A:130
8	BTLA	2AW2	− 6.9	PRO A: 59, GLU A: 57, GLU A: 92, SER A: 44
9	MS4A1	6Y9A	− 6.8	PRO A: 169, PRO A: 160, ASN A:166, GLU A:168, ILE A: 76, TYR A: 77
10	CD79A	7XT6	− 6.4	CYS A:119, ASN A: 73, TYR A:115
11	IGF2R	2V5N	− 8.1	ARG A: 1726, ASP A:1585, VAL A:1584, VAL A:1587, ALA A:1708, PRO A:1707, PHE A:1637, SER A: 1628

**Fig. 9:**
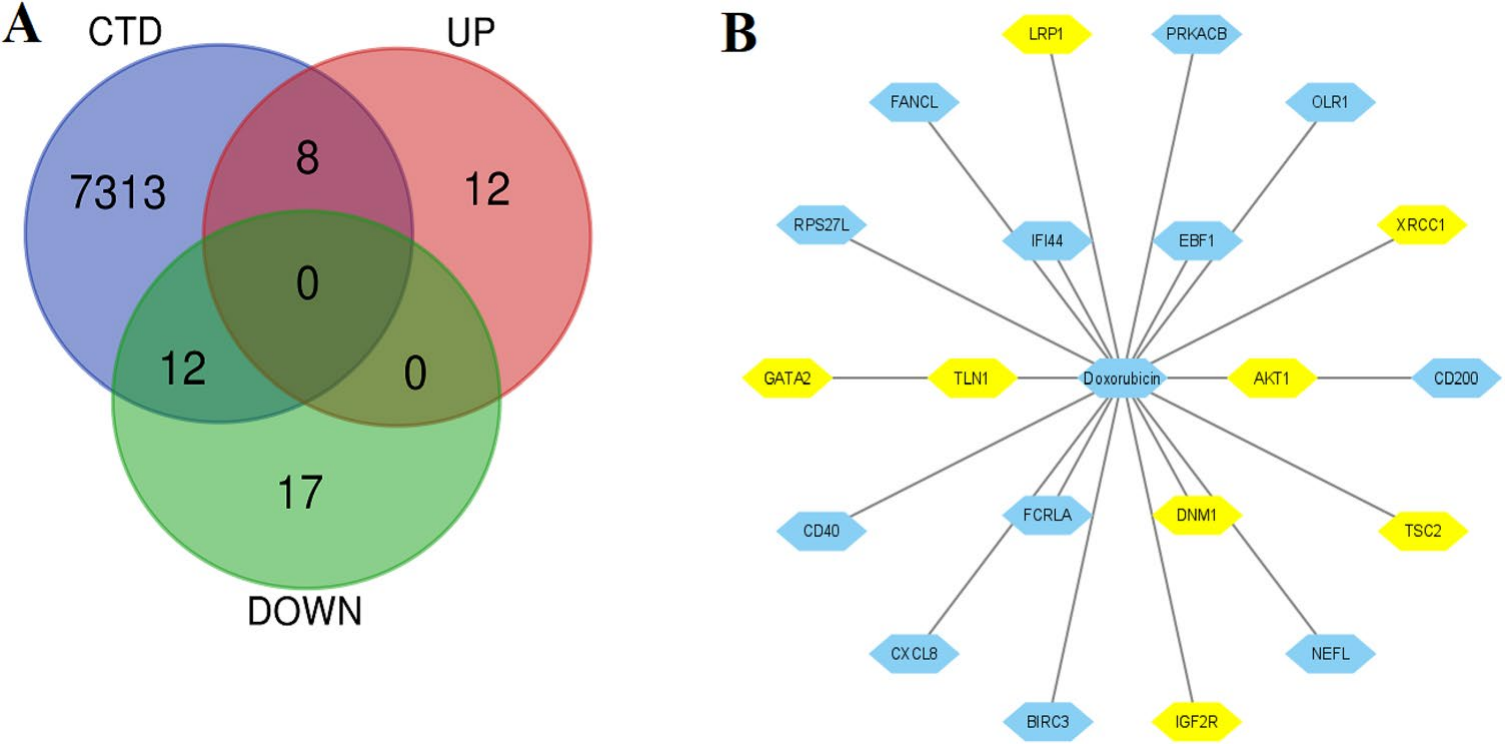
Comparative Toxicogenomics Database (http://ctdbase.org/) identified the interaction of doxorubicin with immune-related hub genes. **A** The Venn diagram identifies 8 upregulated immune-related hub genes, and 12 downregulated immune-related hub genes interacting with doxorubicin. **B** Interaction of immune-related hub genes with doxorubicin. Yellow nodes indicated the upregulated immune-related hub genes, and others stated the downregulated immune-related hub genes

Moreover, we used Comparative Toxicogenomics Database (http://ctdbase.org/) to identify the interaction of doxorubicin with immune-related hub genes. We found that 8 upregulated immune-related hub genes (Fig. 9A), including *XRCC1, LRP1, GATA2, AKT1, TLN1, DNM1, IGF2R, and TSC*2 interacted with doxorubicin and 12 downregulated immune-related hub genes (Fig. 9A), including *OLR1, FANCL, CD40, EBF1, FCRLA, BIRC3, NEFL, CD200, PRKACB, RPS27L, CXCL8,* and *IFI4* interacted with doxorubicin (Fig. 9B).

### Immune-Related Hub Genes Are Deregulated in Cardiomyocytes

**Fig. 10:**
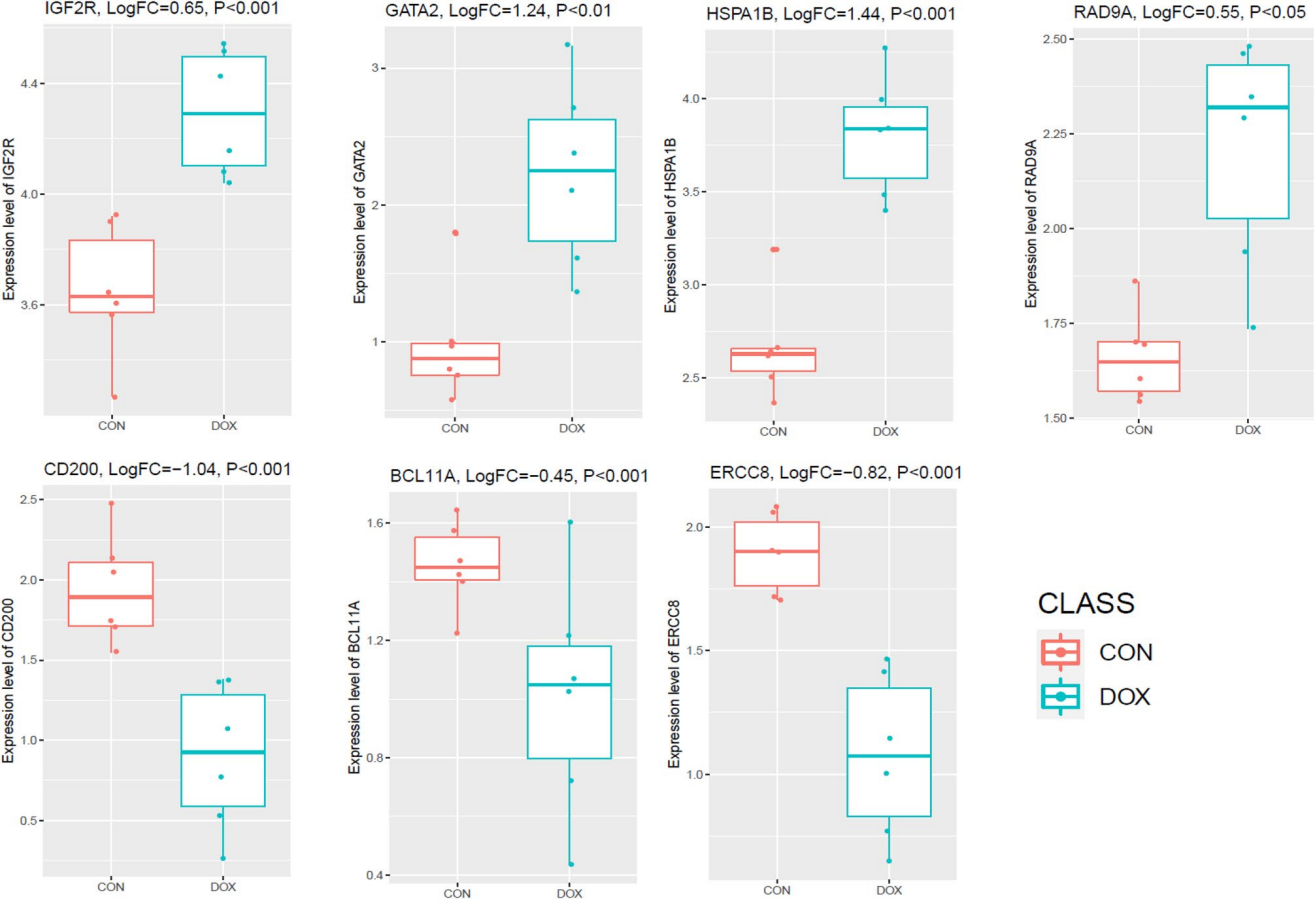
The expression of immune-related genes in human-induced pluripotent stem cell-derived cardiomyocytes (hiPSC-CMs) derived from breast cancer patients who suffered clinical doxorubicin-induced cardiotoxicity. In hiPSC-CMs, *RAD9A, HSPA1B, GATA2,* and *IGF2R* expression levels consistently increase, whereas *CD200, ERCC8,* and *BCL11A* expression levels are downregulated. DOX (*n* = 6): 1 uM doxorubicin for 24 h on gene expression in hiPSC-CM; CON (*n* = 6): 0uM doxorubicin for 24 h on gene expression in hiPSC-CM

We investigated the expression of these immune-related genes in hiPSC-CMs derived from breast cancer patients who suffered clinical doxorubicin-induced cardiotoxicity. We found that *RAD9A, HSPA1B, GATA2,* an*d IGF2R* expression levels are consistently upregulated, and *CD200, ERCC8,* and *BCL11A* are consistently downregulated in hiPSC-CMs (Fig. 10). In contrast, *TLR5* and *IGF2R* are consistently upregulated in heart failure patients not who have breast cancer. It indicates that the doxorubicin induces the dysregulation of hiPSC-CMs derived *RAD9A, HSPA1B, GATA2, IGF2R, CD200, ERCC8,* and *BCL11A* genes that are associated with the pathogenesis of doxorubicin-induced cardiotoxicity in breast cancer patients.

## Discussion

Since gene expression profiles are widely used to identify critical genes and biomarkers for detecting the pathogenesis of diseases [37, 38], we identified the crucial immune-related genes in patients with heart failure and breast cancer who have not been adequately studied using gene expression profiling. Based on the *P*-value < 0.05 [39, 40], the genes aberrantly expressed in women with dox-orubicin-induced heart failure due to breast cancer have been found (Tables 2 and 3, Supplementary Tables S1 and S2). The genes aberrantly expressed in women with doxorubicin-induced heart failure due to breast cancer have been found (Tables 2 and 3). Then, we identified differentially expressed immune-related genes in the doxorubicin-induced heart failure in breast cancer patients (Fig. 2). These aberrantly expressed genes and immune-related transcriptomes are substantially associated with the pathogenesis of breast cancer and heart failure patients. For example, the expression level of *CPA3* elevated after chemotherapy in treating breast cancer tissues [41]. Breast cancer metastasis and basal-like transition are facilitated by HDC-expressing myeloid-derived suppressor cells [42]. *GATA2* was discovered to be a significant epigenetic regulator for G9a in breast cancer, which affects breast cancer cell survival and carcinogenesis [43]. *GATA2* has been identified as a novel gene for coronary artery disease risk, and research on this transcription factor and its downstream effector may reveal a regulatory network crucial for the generation of coronary artery disease [44]. It was reported that *SCN3A* mutations cause early infantile epileptic encephalopathy. It is suggested that a decrease in *TCL1A* levels may contribute to chemo-induced cardiomyopathy by increasing apoptotic sensitivity, decreasing cardiac *MDR1*, and increasing doxorubicin and intracellular free radical concentrations [23]. Moreover, we found that the aberrantly expressed immune-related genes are associated with functional enrichment (Fig. 3). Doxorubicin concurrently activates various controlled cell death mechanisms linked to cardiotoxicity [13]. Several previous studies consistently found that the enriched pathways are associated with breast cancer and heart failure patients. For example, it is widely known that doxorubicin-induced cardiotoxicity comprises apoptosis and autophagy [13, 45]. Chemotherapy with doxorubicin increased cardiac tissue damage linked to impaired lipid metabolism [46]. Both the primary prevention and recurrence of breast cancer are thought to be influenced by immune cells and the cytokine levels released. The functioning of the immune system may be related to the prognosis of cancer and heart failure [47, 48]. Significantly altered gene expression was found in immune cells, as reported by Liyuan Zhang et al. These altered genes are enriched in immune-related pathways and gene ontology terms in breast cancer [49]. These results indicated that doxorubicin causes cardiomyocyte mortality by controlling the signaling and immunological pathways, which could lead to the discovery of new therapeutic medicines and more focused methods for cardiotoxicity studies.

In the PPI, interacted genes were shown to play a role in the occurrence and progression of cardiomyopathy and may serve as targeted therapies and biomarkers for the disease [50]. We identified immune-related crucial genes and their involvement in the extracted gene cluster (Figs. 4 and 5). Previous research investigated how doxorubicin-induced cardiotoxicity in breast cancer patients is connected to immune-related hub genes. Such as Li-Rong Yu et al. discovered the lower CXCL8/IL-8 protein level as a predictive biomarker for doxorubicin-induced cardiotoxicity in breast cancer patients [35]. Furthermore, these immune-related genes are involved with the poor survival prognosis of breast cancer patients (Fig. 6), indicating the importance of these genes in breast cancer patients who progressed to heart failure. Some previous studies consistently reported their roles. For example, *IFIT5* is one of the candidate genes for cardiovascular disease [51]. Through the activation of the NFkB and JNK signaling pathways via *MAP4K1* in cancer cells, *SPIB* functions as a tumor suppressor [52]. Several investigations have revealed that *BTLA* plays a role in various pathogenetic processes, including tumor development, inflammatory diseases, autoimmune disorders, and graft failure [53]. Furthermore, we proved that these prognostic immune-related genes are diagnostic markers in patients with breast cancer and heart failure (Fig. 7). Enfa Zhao et al. predicted potential diagnostic marker genes for cardiovascular diseases and their relevance with immune cell infiltration in this disorder[54]. Interestingly, we found that the protein product of these prognostic immune-related genes (*IFIT5, XCL1, BTLA, MS4A1, CD19, TCL1A, CD83, CD200, FCRLA, CD79A, IGF2R,* and *BIRC3*) have interacted with doxorubicin (Fig. 8) with an appreciable binding affinity (Table 4). Moreover, we discovered numerous aberrantly expressed immune-related genes interacting with doxorubicin (Fig. 9). Doxorubicin interactions with candidate genes cause abnormal gene expression during cancer treatment[55]. Doxorubicin destroys cardiomyocytes by processes similar to those that cause cytotoxicity in cancer cells that it is intended to treat [56]. Finally, we validated that *RAD9A, HSPA1B, GATA2, IGF2R, CD200, ERCC8,* and *BCL11A* genes are consistently dysregulated in the doxorubicin-induced human-induced pluripotent stem cell-derived cardiomyocytes (hiPSC-CMs) derived from breast cancer patients (Fig. 10). However, Li-Rong Yu et al. found some immune response proteins, including CCL23, CCL27, CXCL5, CCL26, CXCL6, GM-CSF, CXCL1, IFN-γ, IL-2, CXCL11, CXCL9, CCL17, CCL25, CXCL1, CCL3, and GDF-1 in doxorubicin-induced cardiotoxicity in breast cancer patients [35]. The magnetic bead-based multiplex immunoassays was used to asses the protein level, but we used data from microarray expression profiling. Hence their results are incongruent with our study. Our patients are different from those in their control samples. We investigated the expression profile in transcriptomic levels, and all tran-scribed genes will not be translated into protein products. However, this result still de-pends on the exact local concentration of the given cytokine, the stage of the disease, and its combination with other combinations of cytokines. Besides, this study has initially demonstrated that inflammation and immunity play essential roles in the early subclin-ical response to DOX-based chemotherapy and that there were significant differences in chemokine profiles or “immune profiles” between the cardiotoxic group and the normal group at each time point after DOX treatment. These immune profiles are associated with inflammatory responses and immune transport and are associated with increased indi-vidual sensitivity to the cardiotoxic effects of DOX in breast cancer patients and may therefore be promising predictive biomarkers of cardiotoxicity. Altogether, doxorubicin-gene interactions may play substantial roles in the cardiotoxicity of breast cancer patients.

Identifying the upregulated and downregulated genes in response to doxorubicin treatment is extremely important, especially for those involved in immunological processes. Based on the results of this research, we make certain assumptions about probable future paths and implications. It is possible to do more mechanistic research to learn how these genes and pathways contribute to the emergence of cardiotoxicity. Developing novel approaches for prospective therapeutic interventions may be possible by better understanding the underlying molecular and cellular systems. Explore whether targeting these genes could enhance patient outcomes by determining their functional involvement in cancer development and cardiotoxicity. Our confirmation of immune-related gene expression in doxorubicin-induced cardiotoxicity in cardiomyocytes obtained from breast cancer patients is impressive. Greater knowledge of the molecular processes resulting in cardiotoxicity in breast cancer patients can be obtained by building on this work by utilizing in vitro models and animal models, allowing for more focused investigations. Insights from our research will be used to determine whether a patient’s unique genetic profile affects how susceptible they are to the cardiotoxicity caused by doxorubicin. Besides, using pharmacogenomics data, treatment strategies could be made more individualized while lowering the possibility of side effects. Finally, investigate the possibility of a combination therapy that uses the immune system to improve the effectiveness of doxorubicin treatment while reducing cardiotoxic effects. In addition, simultaneous consideration of potential interactions between immune-related genes and doxorubicin cardiotoxicity may be a valuable strategy to improve the efficacy of cancer therapy and predict cardiotoxicity.

The primary limitation of our research is that the age of the patients, cancer stage, the severity of heart disease, or co-morbidities that could significantly affect immune-related gene expression were absent in the clinical data of the GSE40447 investigation. Moreover, the limitation of this investigation is the lack of experimental validation in a laboratory setting or our clinical cohort for the important genes and regulatory networks discovered. To apply these findings to treating patients with breast cancer and heart failure, we still need to perform additional experimental and clinical validation, even though our results may offer promising biomarkers for heart failure in breast cancer patients’ diagnosis and treatment as well as therapeutic targets. In addition, to validate these findings and compare them to conventional cardiotoxicity detection methods like echocardiography, prospective clinical research is necessary. More targeted cardioprotective prophylactic and therapeutic approaches are urgently needed to reduce the risk of cardiotoxicity in patients treated with doxorubicin.

## Conclusions

Our findings predicted the interaction of immune-related potential biomarkers with doxorubicin and provided a basis for understanding the mechanism and etiology of the cardiotoxicity produced by doxorubicin in heart failure breast cancer patients. These findings might offer a novel perspective on diagnosing and treating doxorubicin-induced cardiotoxicity in women with breast cancer.

### Supplementary Information

Below is the link to the electronic supplementary material.

Supplementary file1 (TXT 23 KB)

Supplementary file2 (TXT 27 KB)

Supplementary file3 (TXT 375 KB)

Supplementary file4 (TXT 26 KB)

Supplementary file5 (TXT 6 KB)

Supplementary file6 (TXT 5 KB)

Supplementary file7 (TXT 10 KB)

Supplementary file8 (TXT 5 KB)

## Data Availability

We used GSE40447, GSE76314, and TCGA BRCA cohorts for this study. We downloaded the GSE40447 and GSE76314 from the NCBI Gene Expression Omnibus (GEO) database (https://www.ncbi.nlm.nih.gov/geo/). TCGA BRCA cohort was used from the GEPIA webserver.
